# CD8^+^CD28^+^PD1^−^ T Cells as a Prognostic Biomarker in Endometrial Cancer

**DOI:** 10.3390/curroncol32030121

**Published:** 2025-02-21

**Authors:** Yufei Nie, Lin Yang, Yanan Zhang, Hongyan Guo

**Affiliations:** Department of Obstetrics and Gynecology, Peking University Third Hospital, Beijing 100191, China; nyf9055@bjmu.edu.cn (Y.N.)

**Keywords:** endometrial cancer, PD1, CD28, immune microenvironment, prognosis

## Abstract

Endometrial cancer (EC) is an immunogenic tumor, with CD8^+^ T cells playing a pivotal role in antitumor immunity. Overexpression of PD1 suppresses T cell function by inhibiting CD28, a critical co-stimulatory molecule. Classifying CD8^+^ T cells based on PD1 and CD28 expression provides valuable insights into the immune microenvironment of EC. Peripheral blood samples from 120 EC patients and tumor tissue samples from 81 EC patients were analyzed via flow cytometry. CD8^+^ T cells were categorized according to PD1 and CD28 expression, and their associations with clinical characteristics were systematically evaluated. Peripheral CD28^−^/CD8^+^ and PD1^+^/CD8^+^ T cell proportions were significantly associated with several high-risk factors, including deep myometrial invasion, and LVSI, as well as metabolic disorders such as dyslipidemia. Peripheral CD28^+^PD1^−^/CD8^+^ T cells were associated with stage, grade, and LVSI, inversely correlated with age, and reduced in patients with hypertension or dyslipidemia. Tumor-infiltrating CD28^+^PD1^−^/CD8^+^ T cells were associated with tumor grade and LVSI, with multivariate analysis identifying low proportions as an independent predictor of relapse. In summary, CD8^+^CD28^−^ and CD8^+^PD1^+^ T cells are linked to high-risk clinical features in EC, while tumor-infiltrating CD8^+^CD28^+^PD1^−^ T cells serve as a key independent prognostic marker for relapse. Additionally, CD8^+^CD28^−^, CD8^+^PD1^+^, and CD8^+^CD28^+^PD1^−^ T cell proportions in PBMC are closely associated with metabolic disorders, emphasizing their potential as biomarkers for immune and metabolic interactions in EC.

## 1. Introduction

The global incidence of endometrial cancer (EC) has risen significantly over the past three decades, accompanied by an increase in EC-related mortality [1]. Immune cell infiltration has been correlated with patient survival [2], with higher levels of tumor-infiltrating CD8^+^ T cells consistently linked to favorable outcomes [3,4]. This underscores the critical role of cytotoxic T cells in antitumor immunity.

However, CD8^+^ T cells may develop dysfunction within the tumor microenvironment, including exhaustion and senescence. Programmed cell death protein 1 (PD1), a hallmark of T cell exhaustion, is notably expressed in tumor-infiltrating lymphocytes of the POLE and MSI EC subtypes [5]. Exhausted T cells exhibit diminished proliferation and cytotoxicity, enabling tumor immune evasion. Anti-PD1/PD-L1 therapy has shown promise in improving outcomes for patients with advanced or recurrent EC, particularly in MSI subtypes [1].

In addition to exhaustion, T cell senescence represents a pivotal dysfunctional state in cancer. Senescent T cells are characterized by the loss of CD27 and CD28 costimulatory molecules and the acquisition of a senescence-associated secretory phenotype (SASP), which suppresses immunity and facilitates tumor progression [6]. Senescence can be triggered by aging, chronic viral infections, cancer, and metabolic disorders such as obesity and diabetes [7,8,9]—conditions frequently observed in EC patients. This suggests that the high prevalence of these risk factors in EC may exacerbate T cell senescence, impairing immune cytotoxicity. The increased presence of CD8^+^CD28^−^ T cells is associated with poorer prognoses in non-small cell lung and head and neck cancers [10,11] and is more prevalent in the tumor microenvironment than in peripheral circulation in EC patients [12]. Further studies are warranted to explore the relationship between CD8^+^CD28^−^ T cells and clinical outcomes in EC.

The CD28 receptor is crucial for providing costimulatory signals required for naive T cell activation, whereas inhibitory checkpoints like PD1 restrict T cell functionality post-activation, primarily by targeting CD28. This highlights the pivotal role of the CD28/B7 pathway in the efficacy of PD1-targeted therapies [13]. Targeting both T cell senescence and exhaustion within the tumor microenvironment may synergistically enhance antitumor immunity [14]. We hypothesize that CD8^+^CD28^+^PD1^−^ T cells exhibit superior responsiveness to costimulatory signals and reduced sensitivity to inhibitory signals, potentially contributing to improved clinical outcomes in EC.

In this study, we stratified CD8^+^ T cells based on PD1 and CD28 expression in EC patients, correlating these subsets with clinical parameters, including progression-free survival (PFS). Our analysis first examined the expression status of CD28 in EC. Different patterns between CD28 and PD1 determine T cell functionality. These findings provide preliminary insights into the interplay of CD28 and PD1 in the tumor immune microenvironment and suggest the potential for identifying a novel combined prognostic marker of immune status in EC.

## 2. Materials and Methods

### 2.1. Patients and Data Collection

Patients diagnosed with endometrial cancer via pathological examination and who underwent standard staging surgery between August 2021 and September 2024 at Peking University Third Hospital were included. Exclusion criteria were: coexisting malignant tumors, autoimmune diseases, history of immunotherapy, acute or chronic viral infections. Collected data comprised baseline demographics, cancer stage and grade, pathological characteristics (e.g., myometrial invasion, lymphovascular space involvement [LVSI], microcystic, elongated, and fragmented [MELF] pattern), body mass index (BMI), comorbidities, progression-free survival (PFS), and overall survival (OS). Informed consent was obtained from all participants.

### 2.2. Sample Processing

Peripheral blood samples (4 mL) were collected preoperatively in EDTA-coated anticoagulant tubes. Peripheral blood mononuclear cells (PBMCs) were isolated using Ficoll (Cytiva, Uppsala, Sweden)according to the manufacturer’s protocol. Tumor tissues were collected immediately after resection and transported to the laboratory on ice, where they were processed within one hour of collection. The tissue was minced into small pieces on ice and transferred to a 50 mL tube containing 3–4 mL of digestion mix (RPMI containing collagenase IV (Roche, Indianapolis, IN, USA) at a concentration of 1 mg/mL and DNase I (MilliporeSigma, St. Louis, MO, USA) at a concentration of 0.1 mg/mL). The mixture was incubated on a thermal shaker for 45 min at 37 °C and 160 rpm. The resulting cell suspension was then passed through a 70 µm nylon cell strainer (Miltenyi Biotec, Seoul, Republic of Korea) into a 15 mL tube. The cell suspension was mixed with 8–9 mL of red blood cell lysis buffer (BD Biosciences, Franklin Lakes, NJ, USA) and incubated at room temperature for 5 min. PBS was then added to bring the total volume to 15 mL, then the cells were centrifuged for 5 min at 500 g (4 °C). The cells were washed with PBS and resuspended in PBS for staining. An amount of 10 µL of the cell suspension was stained with Trypan Blue and counted using a cell counter.

### 2.3. Flow Cytometry

Both PBMCs and tumor-derived cells were stained with surface marker antibodies (panel 1, as shown in Table 1 and detailed in the Supplementary Table S1) for 15–20 min in the dark at room temperature. Flow cytometry was performed using a CytoFLEX S (Beckman Coulter, Brea, CA, USA), with data analyzed using CytoExpert v2.3 software.

Eleven tumor samples were processed and stained with both surface markers and intracellular markers. Tumor-infiltrating leukocytes were separated via Percoll-based density gradient centrifugation (GE, USA) following the manufacturer’s instructions. A subset of leukocytes (1 × 10^6^ cells) was stained with surface markers (panel 2), while another subset (1 × 10^6^ cells) was cultured with Brefeldin A (5 μg/mL, BioLegend, San Diego, CA, USA) and a cell activation cocktail containing phorbol-12-myristate-13-acetate (PMA) and ionomycin (BioLegend) for 4–6 h. These cells were subsequently stained with surface marker antibodies (panel 3). After fixation and permeabilization using the Intracellular Staining Buffer Kit (BioLegend), intracellular markers were stained with antibodies from panel 4. All antibody details are provided in the Supplementary Table S1. Flow cytometry was conducted using a Spectral Cell Analyzer (SONY ID7000, Sony, Tokyo, Japan), with data analysis performed using FlowJo (v10).

### 2.4. Statistical Analysis

Statistical analyses and graphical visualizations were performed using SPSS (v26.0), GraphPad Prism (v10.1.1), and R software (v 4.3.2). Continuous variables were expressed as mean ± standard deviation. Between-group differences were analyzed using Student’s *t*-test, Mann–Whitney U test, or Kruskal–Wallis rank test for unpaired data, and the Wilcoxon signed-rank test for paired data. Cut-off values for patient stratification (e.g., high- and low-risk groups) were determined using the maximally selected log-rank statistic method in R software. Kaplan–Meier analysis was employed to evaluate PFS, with differences between groups assessed by the log-rank test. Cox proportional hazards regression models were applied for univariate and multivariate analyses. Statistical significance was defined as *p* < 0.05.

## 3. Results

### 3.1. Demographics

We utilized flow cytometry to evaluate the expression of CD28 on CD8^+^ T cells in 120 peripheral blood samples and 81 tumor tissue samples. Additionally, PD-1 expression was assessed in 103 peripheral blood samples and 77 tumor tissue samples. The patient characteristics are summarized in Table 2.

Among the 120 endometrial cancer patients with peripheral blood samples, 76 underwent adjuvant chemotherapy or radiotherapy following surgery. By October 2024, 10 patients experienced disease relapse, including four fatalities. The median PFS was 36.9 months (95% CI: 22.9–46.9), and the median OS was 39.6 months (95% CI: 25.6–47.0).

In the cohort of 81 patients with tumor tissue samples, 64 received adjuvant therapy post-surgery, with 12 experiencing relapse, including six deaths. The median PFS for this group was 14.2 months (95% CI: 5.7–34.2), and the median OS was 14.7 months (95% CI: 6.3–35.6).

### 3.2. Expression of CD28 and PD1 on CD8+ T Cells in PBMC and TME

**Figure 1:**
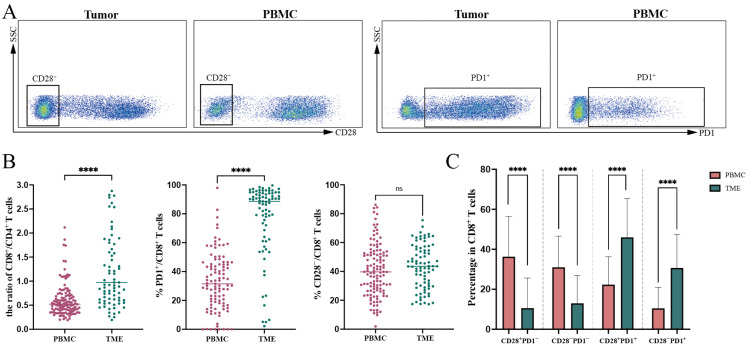
Flow cytometry analysis of CD28 and PD1 expression in CD8^+^ T cells. (**A**) Representative flow cytometry plots display the expression of CD28 and PD1 in CD8^+^ tumor-infiltrating and peripheral lymphocytes from endometrial cancer (EC) patients. (**B**) The ratio of CD8^+^/CD4^+^ T cells and the proportions of PD1^+^/CD8^+^ T cells and CD28^−^/CD8^+^ T cells were compared between PBMC and TME using either *t*-tests or Mann–Whitney U-tests. Data are expressed as mean ± SD. (**C**) Mann–Whitney U-tests were employed to compare the proportions of CD28^+^PD1^−^, CD28^+^PD1^+^, CD28^−^PD1^−^, and CD28^−^PD1^+^ subsets within CD8^+^ T cells between PBMC and TME. Data are expressed as mean ± SD; **** *p* < 0.0001. ns, not significant (*p* > 0.05). PBMC, peripheral blood mononuclear cells; TME, tumor microenvironment.

We gated T cells as shown in Figure 1A to assess the proportions of CD28^−^ and PD1^+^ CD8^+^ T cells within PBMC and the TME, with the results summarized in Table 3. The ratio of CD8^+^ T cells to CD4^+^ T cells was significantly higher in the TME than in PBMC (1.653 vs. 0.678, *p* < 0.0001). Furthermore, the proportion of PD1^+^/CD8^+^ T cells in the tumor microenvironment (TME) was markedly higher compared to PBMC (88.54% vs. 31.48%, *p* < 0.0001), indicating an activated tumor immune environment. However, no significant difference was observed in the proportion of CD28^−^/CD8^+^ T cells between PBMC and TME (40.82% vs. 43.36%, *p* = 0.301) (Figure 1B).

Subsequently, CD8^+^ T cells were categorized based on the combined expression of PD1 and CD28. The proportions of CD28^+^PD1^−^ (4.90% vs. 37.51%, *p* < 0.0001) and CD28^−^PD1^−^ T cells (6.92% vs. 28.50%, *p* < 0.0001) were significantly lower in the TME than in PBMC. In contrast, the proportions of CD28^+^PD1^+^ (47.74% vs. 21.05%, *p* < 0.0001) and CD28^−^PD1^+^ T cells (28.28% vs. 6.78%, *p* < 0.0001) were significantly higher in the TME compared to PBMC (Figure 1C). These findings suggest that although CD8^+^ T cells are enriched and activated in the TME, they predominantly exhibit an exhausted phenotype, which may impair their optimal functionality.

### 3.3. Correlation Between CD28 and PD1 Expression on CD8^+^ T Cells in PBMC and Clinical Characteristics

#### 3.3.1. Association of CD28^−^/CD8^+^ T Cell Proportion in PBMC with Clinical Characteristics

**Figure 2:**
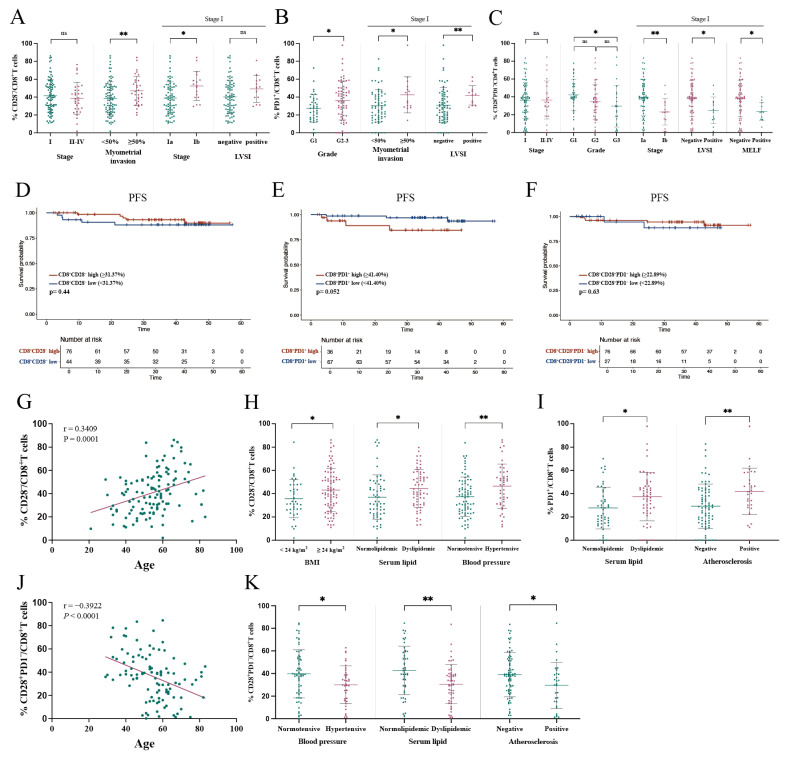
Comparing subsets of peripheral lymphocytes across clinical and pathological risk factors. (**A**) The proportion of CD28^−^/CD8^+^ T cells was compared between patients grouped by various high-risk factors, including including stage I vs. stage II–IV, myometrial invasion <50% vs. ≥50%, stage Ia vs. stage Ib, and LVSI presence vs. LVSI absence in stage I patients. Data are expressed as mean ± SD. (**B**) The proportion of PD1^+^/CD8^+^ T cells was compared between patients grouped by different high-risk factors, including grade 1 vs. grade 2–3, myometrial invasion <50% vs. ≥50% in stage I patients, and LVSI presence vs. LVSI absence in stage I patients. Data are expressed as mean ± SD. (**C**) The proportion of CD28^+^PD1^−^/CD8^+^ T cells was compared between patients grouped by high-risk factors, including stage I vs. stage II–IV, grade 1 vs. grade 2 vs. grade 3, stage Ia vs. stage Ib, LVSI presence vs. LVSI absence in stage I patients, and MELF presence vs. MELF absence in stage I patients. Data are expressed as mean ± SD. (**D**) Kaplan–Meier analysis of PFS in EC patients stratified by the levels of peripheral CD8^+^CD28^−^ T cells. (**E**) Kaplan–Meier analysis for PD1^+^/CD8^+^ T cells and PFS in the same cohort. (**F**) Kaplan–Meier analysis for CD28^+^PD1^−^/CD8^+^ T cells and PFS. (**G**) Pearson correlation analysis of age and the proportion of CD28^−^/CD8^+^ T cells in PBMC. The solid line represents the linear regression fit. (**H**) Comparative analysis of CD28^−^/CD8^+^ T cells in patients with and without metabolic disorders. Data are expressed as mean ± SD. (**I**) Comparative analysis of PD1^+^/CD8^+^ T cells in patients categorized by metabolic characteristics. (**J**) Spearman correlation analysis of age and the proportion of CD28^+^PD1^−^/CD8^+^ T cells in PBMC. The solid line represents the linear regression fit. (**K**) Comparative analysis of CD28^+^PD1^−^/CD8^+^ T cells in patients grouped by metabolic disorders. Data are expressed as mean ± SD. Statistical comparisons were performed using either *t*-tests or Mann–Whitney U-tests. * *p* < 0.05, ** *p* < 0.01. ns, not significant (*p* > 0.05). EC, endometrial cancer; PFS, progression-free survival; PBMC, peripheral blood mononuclear cells; LVSI, lymphovascular space invasion; MELF, microcystic, elongated, and fragmented pattern.

Patients with deep myometrial invasion exhibited a significantly higher proportion of CD28^−^/CD8^+^ T cells in PBMC compared to those without deep invasion (46.98% vs. 38.58%, *p* = 0.025) (Figure 2A). Although no significant difference was observed in the proportion of CD28^−^/CD8^+^ T cells between stage I and stage II–IV patients (41.55% vs. 38.72%, *p* = 0.459), stage Ib patients demonstrated a significantly higher proportion compared to stage Ia patients (52.50% vs. 38.95%, *p* = 0.006) (Figure 2A).

Further subgroup analysis of stage I patients revealed no significant association between the proportion of CD28^−^/CD8^+^ T cells in PBMC and LVSI (Figure 2A). Additionally, we used the maximally selected log-rank statistic method in R software to determine the cut-off value of 31.37% based on the proportion of CD28^−^/CD8^+^ T cells in PBMCs, and stratified patients into high and low subgroups. No significant differences in PFS were observed (Figure 2D).

Subsequently, we analyzed the relationship between CD28 status and metabolic comorbidities. The proportion of CD28^−^/CD8^+^ T cells in PBMC was found to positively correlate with age (*r* = 0.3409, *p* = 0.0001) (Figure 2G). Overweight patients (BMI ≥ 24 kg/m^2^) exhibited a significantly higher proportion of CD28^−^/CD8^+^ T cells in PBMC compared to those with normal BMI (43.17% vs. 35.74%, *p* = 0.037). Similarly, patients with dyslipidemia (44.46% vs. 36.93%, *p* = 0.023) and hypertension (46.47% vs. 37.43%, *p* = 0.008) had higher proportions of CD28^−^/CD8^+^ T cells in PBMC compared to patients with normal lipid levels or blood pressure (Figure 2H).

#### 3.3.2. Association of PD1^+^/CD8^+^ T Cell Proportion in PBMC with Clinical Characteristics

Patients with pathological grades 2–3 exhibited a significantly higher proportion of PD1^+^/CD8^+^ T cells in PBMC compared to those with grade 1 (36.04% vs. 27.32%, *p* = 0.020). Among stage I patients, those with deep myometrial invasion (42.51% vs. 30.19%, *p* = 0.023) or LVSI (42.01% vs. 30.84%, *p* = 0.010) had higher proportions of PD1^+^/CD8^+^ T cells in PBMC compared to those without these high-risk factors (Figure 2B). Stratifying patients into high and low groups based on the proportion of PD1^+^/CD8^+^ T cells in PBMC with a cut-off value of 41.40% did not reveal differences in PFS (Figure 2E).

Additionally, patients with dyslipidemia (37.48% vs. 27.66%, *p* = 0.013) or atherosclerosis (41.89% vs. 29.22%, *p* = 0.003) exhibited higher proportions of PD1^+^/CD8^+^ T cells in PBMC compared to those without these metabolic disorders (Figure 2I).

#### 3.3.3. Association of CD28^+^PD1^−^/CD8^+^ T Cell Proportion in PBMC with Clinical Characteristics

We categorized CD8^+^ T cells into four subpopulations based on the expression of CD28 and PD1, revealing significant correlations between the proportion of CD28^+^PD1^−^/CD8^+^ T cells in PBMC and various clinical characteristics. Patients with pathological grade G3 exhibited a significantly lower proportion of CD28^+^PD1^−^/CD8^+^ T cells in PBMC compared to those with grade G1 (29.58% vs. 42.16%, *p* = 0.020) (Figure 2C). Although no significant difference was observed between stage I and II–IV patients (36.17% vs. 36.54%, *p* = 0.937), a significantly lower proportion was found in stage Ib compared to stage Ia (23.25% vs. 39.24%, *p* = 0.005).

Among stage I patients, those with LVSI (24.85% vs. 38.23%, *p* = 0.032) or MELF pattern invasion (23.68% vs. 38.22%, *p* = 0.024) had lower proportions of CD28^+^PD1^−^/CD8^+^ T cells in PBMC compared to those without these high-risk factors (Figure 2C). However, stratifying EC patients based on the proportion of CD28^+^PD1^−^/CD8^+^ T cells in PBMC using a cut-off value of 22.89% did not reveal differences in PFS (Figure 2F).

The proportion of CD28^+^PD1^−^/CD8^+^ T cells in PBMC was significantly inversely correlated with patient age (*r* = −0.3922, *p* < 0.0001) (Figure 2J). Patients with metabolic comorbidities, including hypertension (30.11% vs. 39.71%, *p* = 0.020), dyslipidemia (30.59% vs. 42.75%, *p* = 0.002), or atherosclerosis (29.54% vs. 39.02%, *p* = 0.030), exhibited significantly lower proportions of CD28^+^PD1^−^/CD8^+^ T cells in PBMC compared to those without these conditions (Figure 2K).

### 3.4. Correlation Between CD28 and PD1 Expression on Tumor-Infiltrating CD8^+^ T Cells and Clinical Characteristics

#### 3.4.1. Lack of Significant Correlation Between Tumor-Infiltrating CD28^−^/CD8^+^ T Cell Proportion and Clinical Characteristics

**Figure 3:**
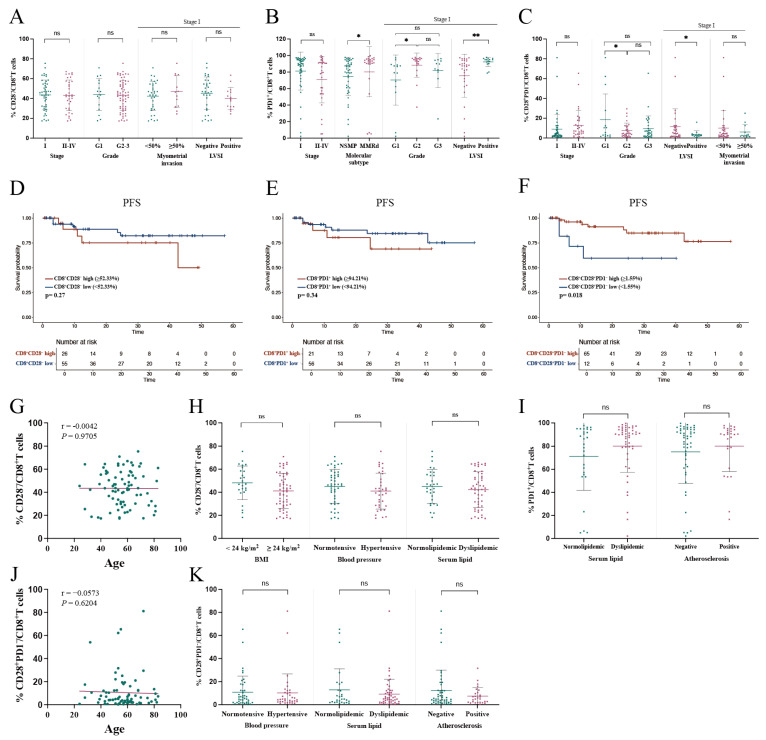
Comparing subsets of tumor-infiltrating lymphocytes across clinical risk factors. (**A**) The proportion of CD28^−^/CD8^+^ T cells was compared between patients grouped by high-risk factors, including including stage I vs. stage II–IV, grade 1 vs. grade 2, myometrial invasion <50% vs. ≥50% in stage I patients, and LVSI presence vs. LVSI absence in stage I patients. Data are expressed as mean ± SD. (**B**) The proportion of PD1^+^/CD8^+^ T cells was compared between patients grouped by high-risk factors, including stage I vs. stage II–IV, NSMP vs. MMRd, grade 1 vs. grade 2 vs. grade 3 in stage I patients, and LVSI presence vs. LVSI absence in stage I patients. Data are expressed as mean ± SD. (**C**) The proportion of CD28^+^PD1^−^/CD8^+^ T cells was compared between patients grouped by high-risk factors, including stage I vs. stage II–IV, grade 1 vs. grade 2 vs. grade 3, LVSI presence vs. LVSI absence in stage I patients, and myometrial invasion <50% vs. myometrial invasion ≥50% in stage I patients. Data are expressed as mean ± SD. (**D**) Kaplan–Meier analysis of PFS in EC patients stratified by the proportions of tumor-infiltrating CD28^−^/CD8^+^ T cells. (**E**) Kaplan-Meier analysis of PFS in the EC patients stratified by the proportions of tumor-infiltrating PD1^+^/CD8^+^ T cells. (**F**) Kaplan-Meier analysis of PFS in the EC patients stratified by the proportions of tumor-infiltrating CD28^+^PD1^−^/CD8^+^ T cells. (**G**) Pearson correlation analysis between age and the proportion of tumor-infiltrating CD28^−^/CD8^+^ T cells. The solid line represents the linear regression fit. (**H**) Comparative analysis of CD28^−^/CD8^+^ T cells in patients grouped by metabolic disorders, such as hypertension, dyslipidemia, and BMI. Data are expressed as mean ± SD. (I) Comparative analysis of PD1^+^/CD8^+^ T cells in patients grouped by metabolic disorders. Data are expressed as mean ± SD. (**J**) Spearman correlation analysis between age and the proportion of CD28^+^PD1^−^/CD8^+^ T cells. The solid line represents the linear regression fit. (**K**) Comparative analysis of CD28^+^PD1^−^/CD8^+^ T cells in patients categorized by metabolic disorders. Data are expressed as mean ± SD. Statistical comparisons were conducted using *t*-tests or Mann–Whitney U-tests. * *p* < 0.05, ** *p* < 0.01. ns, not significant (*p* > 0.05). EC, endometrial cancer; PFS, progression-free survival; LVSI, lymphovascular space invasion; NSMP, no specific molecular profile; MMRd, mismatch repair deficiency; BMI, body mass index.

We investigated the correlation between the proportion of tumor-infiltrating CD28^−^/CD8^+^ T cells and various clinical characteristics, including stage, pathological grade, deep myometrial invasion, and LVSI. No statistically significant differences were observed (Figure 3A).

Additionally, PFS comparisons between patients with high and low proportions of tumor-infiltrating CD28^−^/CD8^+^ T cells using a cut-off value of 52.33% did not reveal any significant differences (Figure 3D).

The proportion of CD28^−^/CD8^+^ T cells in the TME was not correlated with age and showed no significant differences between patients with or without metabolic conditions, including overweight, hypertension, or dyslipidemia (Figure 3G,H).

#### 3.4.2. Association of PD1^+^/CD8^+^ T Cell Proportion in TME with Poor Clinical Characteristics

No significant correlation was found between the proportion of tumor-infiltrating PD1^+^/CD8^+^ T cells and disease stage (Figure 3B). However, patients with microsatellite instability (MSI) exhibited a significantly higher proportion of PD1^+^/CD8^+^ T cells in the TME compared to those with no specific molecular profile (NSMP) (80.22% vs. 74.75%, *p* = 0.018). Among stage I patients, those with pathological grade G2 had a significantly higher proportion of tumor-infiltrating PD1^+^/CD8^+^ T cells compared to grade G1 patients (88.32% vs. 70.32%, *p* = 0.011). Similarly, patients with LVSI had a higher proportion of PD1^+^/CD8^+^ T cells compared to those without LVSI (92.2% vs. 75.6%, *p* = 0.006) (Figure 3B).

No significant correlation was observed between the proportion of tumor-infiltrating PD1^+^/CD8^+^ T cells and PFS using a cut-off value of 94.21% (Figure 3E). Additionally, no statistical differences were found in the proportion of tumor-infiltrating PD1^+^/CD8^+^ T cells between patients with or without dyslipidemia or atherosclerosis (Figure 3I).

#### 3.4.3. Association of CD28^+^PD1^−^/CD8^+^ T Cell Proportion in TME with Clinical Characteristics

Further analysis categorized tumor-infiltrating CD8^+^ T cells into four subpopulations based on CD28 and PD1 expression, revealing associations with clinical characteristics. Patients with pathological grade G2 exhibited a significantly lower proportion of tumor-infiltrating CD28^+^PD1^−^/CD8^+^ T cells compared to grade G1 patients (7.59% vs. 18.69%, *p* = 0.019), while no statistical difference was observed between grades G3 and G1 (9.56% vs. 18.69%, *p* = 0.052). No significant differences were identified between stage I and II–IV patients. However, among stage I patients, those with LVSI had a significantly lower proportion of tumor-infiltrating CD28^+^PD1^−^/CD8^+^ T cells compared to those without LVSI (3.54% vs. 11.66%, *p* = 0.023) (Figure 3C).

Patients stratified into high and low groups based on the proportion of tumor-infiltrating CD28^+^PD1^−^/CD8^+^ T cells using a cut-off value of 1.55% demonstrated that the high-proportion group had prolonged PFS (*p* = 0.018) (Figure 3F). Multivariate analysis further indicated that the proportion of CD28^+^PD1^−^/CD8^+^ T cells in the TME was an independent predictor of relapse (HR = 0.099, 95% CI: 0.022–0.446, *p* = 0.003) (Table 4).

Analysis of metabolic disorders, including hypertension, dyslipidemia, and atherosclerosis, showed no significant correlation with the proportion of tumor-infiltrating CD28^+^PD1^−^/CD8^+^ T cells (Figure 3K). Similarly, Spearman correlation analysis between age and the proportion of CD28^+^PD1^−^/CD8^+^ T cells showed no significant association (Figure 3J).

### 3.5. Functional Activity of CD8^+^ T Cells in TME

**Figure 4:**
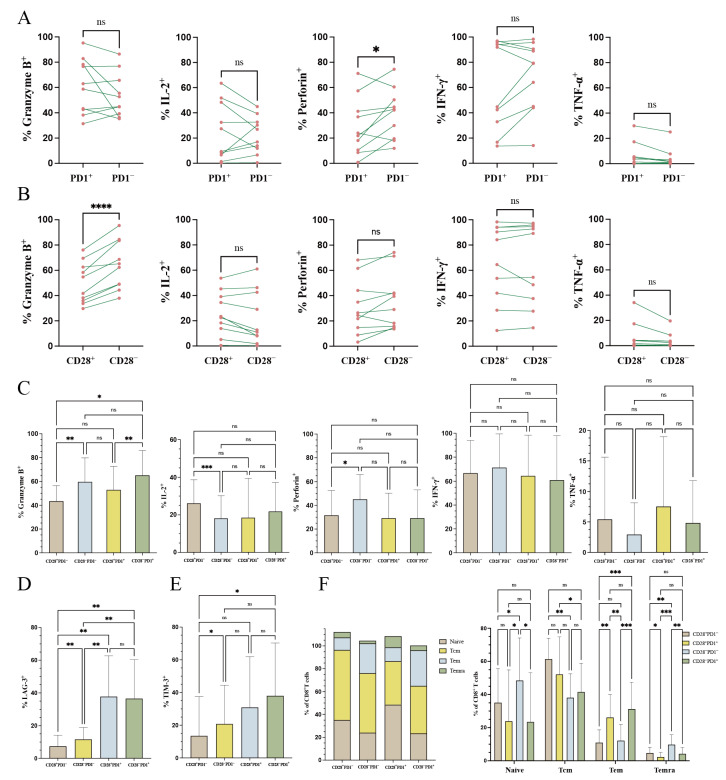
Analysis of cytotoxic molecules and inhibitory receptors in tumor-infiltrating CD8^+^ T cell subsets. (**A**–**C**) Cumulative data illustrating the production of granzyme B, IL-2, perforin, IFN-γ, and TNF-α by tumor-infiltrating CD8^+^ T cell subpopulations following stimulation with PMA and BFA for 5 h (*n* = 11). Data are expressed as mean ± SD. (**D**,**E**) Proportions of LAG-3 and TIM-3 expression within tumor-infiltrating CD8^+^ T cell subpopulations. (**F**) Distribution of naïve (CD45RA^+^CCR7^+^), central memory (CD45RA^−^CCR7^+^, Tcm), effector memory (CD45RA^−^CCR7^−^, Tem), and terminal effector memory (CD45RA^+^CCR7^−^, Temra) subsets among the four CD8^+^ T cell subgroups stratified by CD28 and PD1 expression. Data are expressed as mean ± SD. Statistical significance was assessed using the Wilcoxon matched-pairs signed rank test and one-way ANOVA. *p*-values were adjusted with the Bonferroni correction, and significance levels are denoted as follows: * *p* < 0.05, ** *p* < 0.01, *** *p* < 0.001, and **** *p* < 0.0001. ns, not significant (*p* > 0.05). Tcm, central memory T cells; Tem, effector memory T cells; Temra, terminal effector memory T cells.

To investigate the effects of CD28 and PD1 expression on CD8^+^ T cell functionality, we analyzed tumor-infiltrating T cells extracted from 11 EC patients, focusing on their cytokine expression profiles. Our results demonstrated a significantly lower proportion of perforin-positive CD8^+^PD1^+^ T cells compared to CD8+PD1- T cells (29.06% vs. 39.43%, *p* = 0.036) (Figure 4A). Similarly, the granzyme B expression rate was markedly higher in CD8^+^CD28^−^ T cells than in CD8^+^CD28^+^ T cells (63.96% vs. 50.04%, *p* < 0.0001) (Figure 4B). The positive rates of granzyme B (43.48% vs. 59.69%, *p* = 0.003) and perforin (31.75% vs. 45.12%, *p* = 0.029) were significantly lower in CD8^+^CD28^+^PD1^−^ T cells compared to CD8^+^CD28^−^PD1^−^ T cells. However, CD8^+^CD28^+^PD1^−^ T cells exhibited a significantly higher IL-2 positivity rate than CD8^+^CD28^−^PD1^−^ T cells (25.78% vs. 19.79%, *p* = 0.004) (Figure 4C).

Notably, the proportion of LAG-3^+^ T cells in CD8^+^CD28^+^PD1^−^ T cells (7.42%) was significantly lower than in CD8^+^CD28^−^PD1^−^ (11.64%, *p* = 0.008), CD8^+^CD28^+^PD1^+^ (37.79%, *p* = 0.001), and CD8^+^CD28^−^PD1^+^ T cells (36.52%, *p* = 0.001) (Figure 4D). Similarly, the proportion of TIM-3^+^ T cells in CD8^+^CD28^+^PD1^−^ T cells (13.51%) was significantly lower than in CD8^+^CD28^−^PD1^−^ (20.77%, *p* = 0.0235) and CD8^+^CD28^−^PD1^+^ T cells (38.01%, *p* = 0.0351) (Figure 4E).

Using CD45RA and CCR7 expression, we categorized T cells into naïve (CD45RA^+^CCR7^+^), central memory (CD45RA^−^CCR7^+^, Tcm), effector memory (CD45RA^−^CCR7^−^, Tem), and terminal effector memory (CD45RA^+^CCR7^−^, Temra) subsets. Our analysis revealed that CD8^+^CD28^+^PD1^−^ T cells predominantly consisted of Tcm, with a significantly higher proportion of Tcm compared to CD8^+^CD28^−^PD1^−^ T cells (61.41% vs. 38.07%, *p* = 0.0056) (Figure 4F).

## 4. Discussion

Our study revealed that the proportion of peripheral CD8^+^CD28^−^ T cells was closely associated with deep myometrial invasion, a factor linked to poor prognosis and critical for staging early-stage EC [15]. This proportion was higher in stage Ib compared to stage Ia. However, no correlation was observed between the proportion of peripheral CD8^+^CD28^−^ T cells and relapse. Additionally, the proportion of tumor-infiltrating CD8^+^CD28^−^ T cells was not significantly associated with clinical characteristics or prognosis. To date, no prior studies have examined the role of CD28 expression in EC prognosis, though it has been implicated in stage and relapse in head and neck, lung, and breast cancers [10,11,16]. Further research is required to elucidate the impact of CD8^+^CD28^−^ T cells on EC prognosis.

Our findings also demonstrated an association between PD1 expression and high-risk clinical characteristics, although no direct correlation with relapse was observed. Patients with grades 2–3 tumors exhibited a higher proportion of peripheral CD8^+^PD1^+^ T cells than those with grade 1 tumors. Moreover, stage I patients with deep myometrial invasion or LVSI showed elevated peripheral CD8^+^PD1^+^ T cell proportions. Within the TME, CD8^+^PD1^+^ T cell proportions were associated with grade and LVSI, factors that significantly influence EC recurrence. These findings suggest that PD1 is involved in tumor progression [1], corroborating its association with a more robust immune response in MSI tumors. Our study aligns with previous reports showing that MSI tumors exhibit a higher PD1^+^ proportion and respond more effectively to immunotherapy [5,17].

A pan-cancer analysis has highlighted the prognostic significance of PD1, with its effects varying across tumor types [18]. Exhausted CD8^+^ T cells exhibit heterogeneity, with stem-like progenitor exhausted cells retaining effector capabilities, while terminally differentiated cells, characterized by high PD1 expression and co-inhibitory molecules, display impaired functionality [19]. For example, in lung cancer, terminally exhausted T cells expressing HPK1^+^PD1^+^TIM-3^+^CD8^+^ are associated with poor prognosis [20], while glioblastoma studies suggest a link between low CD8^+^CD103^+^PD1^+^TIM3^+^ Trm cells and prolonged survival [21]. Such heterogeneity limits the utility of PD1 as a standalone prognostic marker, underscoring the need for further differentiation within PD1^+^ T cell subpopulations.

Studies in lung cancer have shown that CD8^+^CD28^+^PD1^−^ T cells exhibit a more naive phenotype [22], although research on this T cell subset in relation to tumors is currently limited. Our study identified a strong correlation between the proportion of CD8^+^CD28^+^PD1^−^ T cells and clinical characteristics in EC patients. This subset was associated with multiple high-risk features, with lower proportions observed in stage Ib compared to stage Ia, in grade 3 tumors compared to grade 1, and in stage I patients with LVSI. Within the TME, CD8^+^CD28^+^PD1^−^ T cell proportions were independently predictive of relapse. This subset predominantly consisted of central memory T (Tcm) cells, characterized by superior persistence and antitumor activity compared to effector memory T (Tem) or effector T (Teff) cells. Our findings suggest that CD8+CD28^+^PD1^−^ T cells may play a critical role in antitumor immunity and serve as a valuable predictor of relapse risk in EC.

The interplay between CD28 and PD1 pathways also highlights therapeutic implications. PD1 inhibits T cell function primarily through CD28 suppression, with anti-PD1 therapy efficacy relying on CD28 molecules [13,23,24]. Circulating CD28^+^ T cell proportions correlate positively with responses to anti-PD1/PD-L1 therapy and prognosis but are also associated with adverse immunotherapy events [25]. Enhancing CD28 co-stimulatory signals alongside PD1 inhibition may amplify T cell-mediated antitumor effects [26], as demonstrated in melanoma models [27].

Although CD8^+^CD28^+^PD1^−^ T cells exhibited reduced granzyme B and perforin expression, they demonstrated higher IL-2 positivity, lower LAG-3 and TIM-3 expression, and a greater proportion of central memory (Tcm) cells. Previous studies have shown that CD28^−^ T cells secrete higher levels of cytotoxic factors, including perforin, granzyme, hemolytic granules, and pro-inflammatory cytokines such as TNF-α [9]. In lung cancer, CD28^−^PD1^+^ and CD28^−^PD1^−^ T cells displayed enhanced secretory capabilities for granzyme B, IFN-γ, and TNF-α, whereas CD28^+^PD1^−^ and CD28^+^PD1^+^ T cells exhibited diminished secretory functions, consistent with our findings. IL-2, a key growth factor for tumor-infiltrating lymphocytes [28], suggests that CD8^+^CD28^+^PD1^−^ T cells possess superior proliferative potential. Additionally, central memory (Tcm) T cells have been reported to exhibit greater persistence and antitumor immunity compared to effector memory (Tem) and effector T (Teff) cells [29]. These characteristics align with the enhanced proliferative and antitumor functions of CD8^+^CD28^+^PD1^−^ T cells, potentially explaining their association with prolonged PFS in EC. Further investigation into the antitumor functions of CD8^+^CD28^+^PD1^−^ T cells is warranted.

Finally, we observed a link between peripheral CD8^+^ T cell subsets and metabolic disorders. The proportion of CD8^+^CD28^−^ and CD8^+^PD1^+^ T cells was positively associated with dyslipidemia, while CD8^+^CD28^+^PD1^−^ T cells were inversely associated. These findings suggest that lipid metabolism may influence T cell phenotypes, with implications for immune status in EC patients, who often present with obesity and metabolic comorbidities.

This study has several limitations: (1) A deeper functional analysis of T cells was not conducted. (2) The cohort’s small sample size with endpoint events precluded overall survival analysis. (3) The follow-up period was relatively short.

## 5. Conclusions

The proportions of CD8^+^CD28^−^ and CD8^+^PD1^+^ T cells are associated with high-risk factors in EC, while tumor-infiltrating CD8^+^CD28^+^PD1^−^ T cells independently predict relapse. These subsets are also linked to metabolic disorders, highlighting their potential as biomarkers and therapeutic targets in EC.

## Figures and Tables

**Table 1:** List of markers in antibody panels.

Panel Name	Target Markers
Panel 1	CD4, CD8, CD28, PD1
Panel 2	PD1, LAG-3, TIM-3, CD45, CD3, CD4, CD8, CCR7, CD45RA, CD28
Panel 3	CD4, CD8, CD28, PD1
Panel 4	IFN-γ, Granzyme B, IL-2, TNF-α, Perforin

**Table 2:** The clinical characteristics of endometrial cancer patients.

Characteristic	PBMC (n = 120)	Tumor (n = 81)
Age(years)	54.6 ± 12.2	56.4 ± 12.8
FIGO stage		
I	89 (74.2%)	47 (58.0%)
II	6 (5.0%)	6 (7.4%)
III	19 (15.8%)	20 (24.7%)
IV	6 (5.0%)	8 (9.9%)
Grade		
G1	41 (34.2%)	16 (19.8%)
G2	54 (45.0%)	33 (40.7%)
G3	25 (20.8%)	32 (39.5%)
Histotype		
Endometrioid	106 (88.3%)	71 (87.7%)
Non-endometrioid	14 (11.7%)	10 (12.3%)
Molecular subtype		
POLEmut	2 (1.7%)	1 (1.2%)
MMRd	16 (13.3%)	24 (29.6%)
NSMP	91 (75.8%)	48 (59.3%)
p53abn	10 (8.3%)	7 (8.6%)
Metabolic disorders		
Hypertension	45 (37.5%)	36 (44.4%)
Diabetes mellitus	22 (18.3%)	21 (25.9%)
Atherosclerosis	31 (25.8%)	27 (33.3%)
Fatty liver	59 (49.2%)	33 (40.7%)
Hyperlipidemia	62 (51.7%)	51 (63.0%)
Obesity	36 (30.0%)	26 (32.1%)
Vital status at last follow-up		
Tumor-Free Survival	110 (91.7%)	69 (85.2%)
Relapse	10 (8.3%)	12 (14.8%)
Deceased	4 (3.3%)	6 (7.4%)

**Table 3:** Distribution of CD8^+^T lymphocyte subpopulation in different cohort.

Markers	PBMC (%)	Tumor (%)
CD8^+^	36.48 ± 12.25	51.81 ± 17.61
CD28^+^/CD8^+^	59.18 ± 18.19	56.64 ± 15.18
CD28^−^/CD8^+^	40.82 ± 18.19	43.36 ± 15.18
PD1^+^/CD8^+^	31.48 (18.01, 45.16)	88.54 (66.33, 94.25)
CD28^+^PD1^+^/CD8^+^	21.05 (11.24, 33.35)	47.74 (33.36, 61.03)
CD28^−^PD1^+^/CD8^+^	6.78 (3.28, 15.03)	28.28 (19.33, 42.24)
PD1^−^/CD8^+^	68.57 (54.88, 82.01)	11.93 (5.76, 31.36)
CD28^+^PD1^−^/CD8^+^	37.51 (22.57, 49.06)	4.90 (2.12, 12.26)
CD28^−^PD1^−^/CD8^+^	28.50 (18.47, 40.33)	6.92 (3.20, 18.54)

Numbers were presented as mean ± standard deviation; PD1 was tested in 77 tumor tissue and 103 PBMC samples.

**Table 4:** Univariate and multivariate analysis for PFS in the tumor cohort (n = 77).

Variable	Univariate Analysis			Multivariate Analysis		
	HR	95% CI	*p*	HR	95% CI	*p*
Stage	3.395	2.120–5.438	<0.001	2.148	1.227–3.760	0.007
Myometrium invasion ≥ 50%	9.158	2.904–28.883	<0.001	10.230	2.092–50.024	0.004
%CD28^+^PD1^−^/CD8^+^ TILs	0.245	0.069–0.871	0.030	0.099	0.022–0.446	0.003
Grade	4.425	1.901–10.304	<0.001			0.576
LVSI	4.945	1.748–13.988	0.003			0.846

Abbreviations: TILs: tumor-infiltrating lymphocytes, LVSI: lymphovascular space invasion, HR: hazard ratio, CI: confidential interval. Multivariate analysis was performed by the Cox multivariate proportional hazard regression model with stepwise manner.

## Data Availability

The data supporting the reported results are not publicly available due to privacy restrictions. However, they may be available from the corresponding author upon reasonable request and with appropriate ethical approvals.

## Supplementary Materials

The following supporting information can be downloaded at: https://www.mdpi.com/article/10.3390/curroncol32030121/s1, Table S1: Markers included in the antibody panels.

## Abbreviations

The following abbreviations are used in this manuscript:

EC	Endometrial cancer
PD1	Programmed cell death protein 1
LVSI	Lymphovascular space invasion
MSI	Microsatellite Instability
SASP	Senescence-associated secretory phenotype
MELF	Microcystic, elongated, and fragmented pattern
BMI	Body mass index
PFS	Progression-free survival
OS	Overall survival
PBMC	Peripheral blood mononuclear cells
PMA	Phorbol-12-myristate-13-acetate
TME	Tumor microenvironment
NSMP	No specific molecular profile
Tcm	Central memory T cells
Tem	Effector memory T cells
Temra	Effector memory T cells re-expressing CD45RA
