# Supplementary material for: CD8+CD28+PD1− T Cells as a Prognostic Biomarker in Endometrial Cancer
Source: Curr Oncol. 2025 Feb 21;32(3):121. doi: 10.3390/curroncol32030121 (PMC11940843; doi:10.3390/curroncol32030121)
Supplement: Supplementary file 1 [file curroncol-32-00121-s001.zip › curroncol-3456481-supplementary.pdf]

**Table S1.** Markers included in the antibody panels.

| | Antibody | Manufacturer |
|---|---|---|
| **Panel 1** | APC-Cy7 anti-human CD4 | Biolegend |
| | PerCP-Cy5.5 anti-human CD8 | Biolegend |
| | PE-Cy7 anti-human CD28 | Biolegend |
| | PE anti-human PD1 | Biolegend |
| **Panel 2** | BV605 anti-human PD1 | Biolegend |
| | BV650 anti-human LAG-3 | Biolegend |
| | BV421 anti-human TIM-3 | Biolegend |
| | APC anti-human CD45 | Biolegend |
| | APC-cy7 anti-human CD3 | Biolegend |
| | PE anti-human CD4 | Biolegend |
| | PerCP-Cy5.5 anti-human CD8 | Biolegend |
| | PE-CF594 anti-human CCR7 | Biolegend |
| | BV510 anti-human CD45RA | Biolegend |
| | PE-Cy7 anti-human CD28 | Biolegend |
| **Panel 3** | PE anti-human PD1 | Biolegend |
| | APC-cy7 anti-human CD4 | Biolegend |
| | PerCP-cy5.5 CD8 | Biolegend |
| | PE-Cy7 anti-human CD28 | Biolegend |
| **Panel 4** | FITC anti-human IFN-$\gamma$ | Biolegend |
| | APC anti-human Granzyme B | Biolegend |
| | BV605 anti-human IL-2 | Biolegend |
| | PE-CF594 anti-humanTNF-$\alpha$ | Biolegend |
| | BV510 anti-human Perforin | Biolegend |
